# MR Imaging of SLAP Lesions

**DOI:** 10.2174/1874325001812010314

**Published:** 2018-07-31

**Authors:** Robert D. Boutin, Richard A. Marder

**Affiliations:** 1 Department of Radiology, UC Davis School of Medicine, 4860 Y St., Suite 3100, Sacramento, CA 95817, USA; 2 Department of Orthopaedic Surgery, UC Davis School of Medicine, 4860 Y St., Suite 3800, Sacramento, CA 95817, USA

**Keywords:** SLAP tear, MRI, Superior labrum, Biceps anchor, MR arthrography, Intra-articular

## Abstract

**Background:**

SLAP lesions of the shoulder are challenging to diagnose by clinical means alone. Interpretation of MR images requires knowledge of the normal appearance of the labrum, its anatomical variants, and the characteristic patterns of SLAP lesions. In general, high signal extending anterior and posterior to the biceps anchor is the hallmark of SLAP lesions. Common diagnostic criteria for a SLAP lesion by MR or MR arthrography include the following: presence of a laterally curved, high signal intensity in the labrum on a coronal image, multiple or branching lines of high signal intensity in the superior labrum on a coronal image, full-thickness detachment with irregularly marginated high signal intensity and/or separation >2 mm on conventional MRI or 3 mm on MR arthrography between the labrum and glenoid on a coronal image, and a paralabral cyst extending from the superior labrum.

**Methods:**

MR diagnosis of SLAP tears may be improved with provocative maneuvers, such as longitudinal traction of the arm or positioning of the shoulder in abduction and external rotation during imaging. The use of intra-articular contrast distends the joint similar to what occurs during arthroscopy and forced diffusion under the labrum may improve the ability to detect SLAP lesions that might not be seen with standard MR. Improved diagnostic accuracy for SLAP tears is seen with 3-T compared with 1.5-T MR imaging, with or without intra-articular contrast material.

**Conclusion:**

Regardless of MR findings, however, physicians should be cautious when recommending surgery in the patient with a vague clinical picture. The patient’s history, physical exam, and imaging evaluation all should be considered together in making the decision to proceed with surgery.

## 1 INTRODUCTION

SLAP lesions of the shoulder are challenging to diagnose by clinical means alone [1]. Injury occurs from inferior traction on the shoulder, as well as excessive tension on and twisting of the Long Head of Biceps tendon (LHB) at its insertion along the superior labrum that occurs during the cocking motion of throwing [2, 3]. These mechanisms of injury are also associated with a myriad of other shoulder conditions including rotator cuff tendinopathy, bicipital tendinopathy and subluxation, internal impingement, and glenohumeral instability. Thus, history, while helpful, only suggests the diagnosis of a SLAP lesion.

Standard shoulder physical examination of the shoulder may be normal unless instability is present. Specialized exam tests have been developed that help the LHB insertion to assist in the clinical diagnosis. Such provocative maneuvers include the active compression test, biceps load test II, Speed’s test, the resisted supination external rotation test, and the dynamic labral shear test [4-7]. Variable success in predicting a SLAP lesion has been reported with these tests in systematic reviews [8, 9], and one rigorous Level I study has shown poor correlation with arthroscopy [10]. When used in combination, the accuracy to diagnosis a SLAP lesion by physical exam may be improved [11-13].

Correct diagnosis of SLAP lesions is important because the ability to confidently predict that a patient does not have a SLAP lesion can prevent an arthroscopy that simply discovers a normal superior labrum. Furthermore, the ability to accurately diagnose the type of SLAP lesion allows pre-operative planning, for not only the surgical procedure, but also post-operative rehabilitation, which can be prolonged in certain cases. Imaging is a potentially helpful tool in diagnosing a SLAP tear pre-operatively. Both MR and MR arthrography have been used with varying degrees of success [14-18].

## 2 IMAGING

**Fig. (1):**
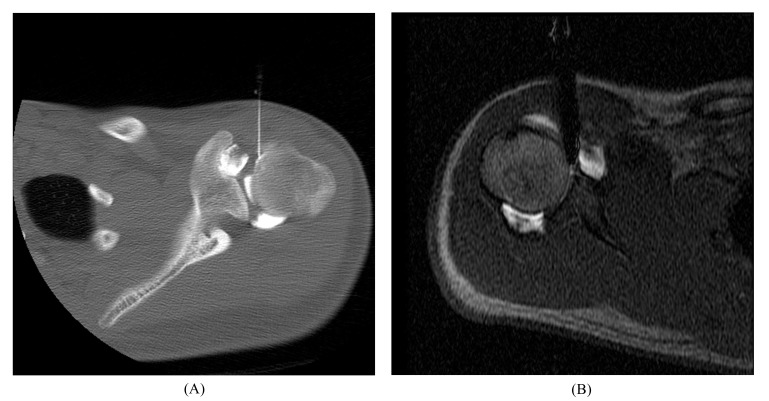
Arthrogram procedure. Anterior approach of needle for image-guided glenohumeral joint injection of contrast material using (**A**) CT and (**B**) MRI.

At our institution, routine MR of the shoulder is performed using a 1.5–Tesla (T) or 3-T scanner to obtain Fast Spin-Echo (FSE) proton-density and fat-suppressed T2-weighted images in the axial, coronal oblique, and sagittal oblique planes. Images in the axial and coronal oblique planes are most useful for the evaluation of SLAP lesions. For MR arthrography, percutaneous injection of diluted gadolinium (12 mL, 1:200 dilution) into the glenohumeral joint is performed using an anterior approach. Radiologists usually confirm intra-articular injections fluoroscopically, although sonography, CT, or MRI also may be used (Fig. **1**). MR scanning begins within 30 minutes of injection, and commonly includes FSE fat-suppressed T1-weighted images in all three planes (which highlight any contrast material filling labral tear), as well as FSE coronal fat-suppressed T2-weighted images (which show any paralabral cyst) and sagittal T1-weighted images (which show fatty muscle atrophy associated with a chronic cuff tear or muscle denervation). Detailed, updated standard of practice imaging protocols are posted online [19].

## 3 IMAGING INTERPRETATION

**Fig. (2):**
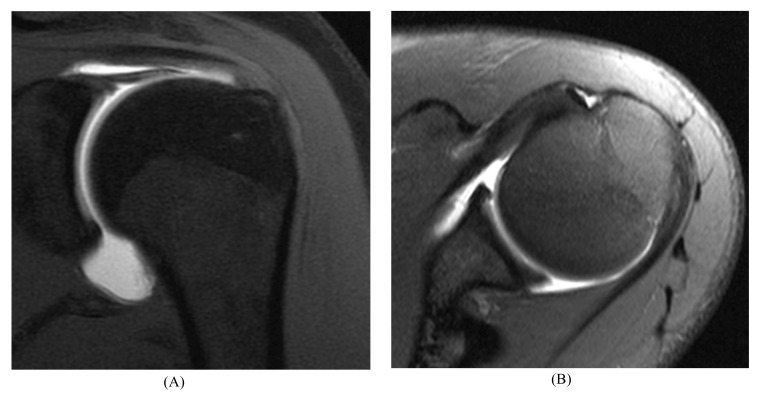
Normal labrum. 14 year-old male athlete with an unequivocally normal labrum. (**A**) Coronal and (**B**) axial fat-suppressed T1-weighted MR arthrogram images show normal low signal intensity fibrocartilage; no contrast material extends into the labrum or into the chondrolabral junction.

Correct interpretation of MR images requires knowledge of the normal appearance of the labrum, its anatomical variants, and the characteristic patterns of SLAP lesions [20]. The fibrocartilaginous labrum most often has a triangular structure, but it may change shape dynamically with traction from the capsule or glenohumeral ligaments (*e.g.,* appear round or flattened). Regardless, if there is no high signal either at the labral–chondral junction or through the labrum, the labrum should be graded as normal [21] (Fig. **2**).

**Fig. (3):**
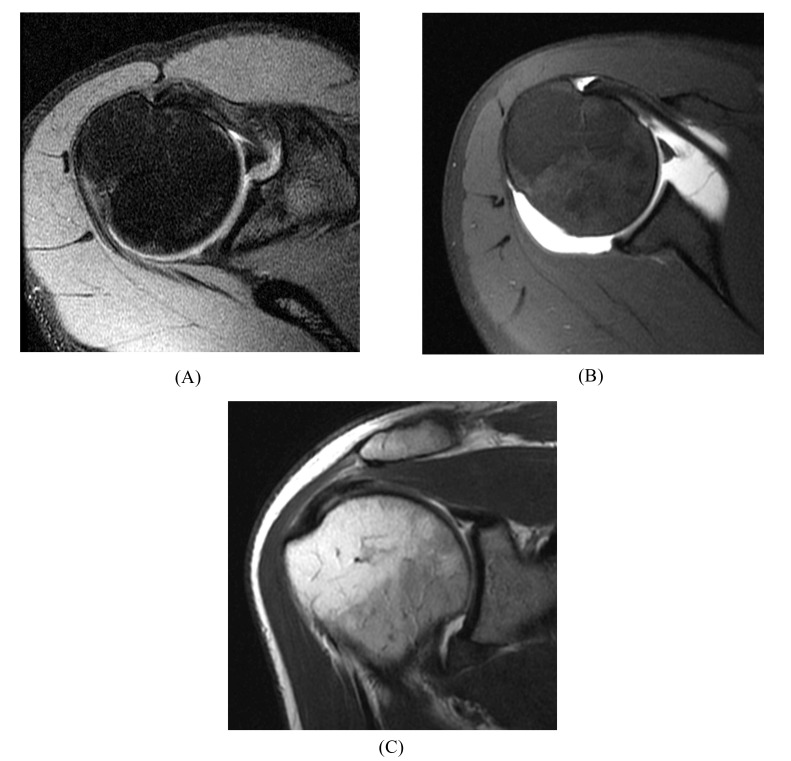
Buford complex. (**A**) Axial fat-suppressed T2-weighted image of a 39 year-old male athlete with an absent anterosuperior labrum and a thickened, cord-like middle glenohumeral ligament. (**B**) Axial fat-suppressed T1-weighted MR arthrogram image in a 44 year-old orthopaedist, a female athlete with a Buford complex. (**C**) Coronal T1-weighted MR arthrogram image in the same shoulder shows a superior labral tear.

Anatomic variations in the labrum most commonly occur in the anterior-superior quadrant (between 12 and 3 o’clock), including the Buford complex, sublabral sulcus, and sublabral foramen [22-25]. A Buford complex (Fig. **3**) is a distinct congenital condition that occurs in approximately 1 to 7% of normal shoulders [25]. This anatomic variant is defined by an absent upper anterior quadrant of the labrum and a thickened, cord-like middle glenohumeral ligament that inserts directly into the superior labrum. Interestingly, although the Buford complex is regarded as an anatomic variant, recent literature indicates that it may predispose patients to the development of SLAP lesions [26], perhaps due to altered biomechanics [27].

The sublabral sulcus (also referred to as a sublabral recess) and sublabral foramen are frequent anatomic variations in which a segment of the labrum is not attached to the underlying glenoid. The sublabral sulcus is particularly important; a partially unattached labrum with some form of a superior recess is reported in up to 75% of patients without a superior labral tear [28]. This occurs at the apex of the superior labrum, but classically does not extend posteriorly to the LHB attachment. The depth of the recess is variable, but typically less than 2 mm [25].

Beginning with the classic work by DePalma [29], authors have suggested that the detachment at the chondrolabral junction is an age-dependent degenerative phenomenon because superior recesses are not present at birth, and the incidence and depth of recesses increases with age [29-31]. Indeed, the high prevalence of superior chondrolabral detachment and marginal benefits of SLAP repair after patients reach the age of 40 to 45 years should be considered when making treatment decisions [27, 32]. Although, a sublabral sulcus typically appears as a cleft of smooth, medially curved increased signal at the chondrolabral junction that does not extend posterior to the biceps anchor, these normal variants reportedly can extend posterior to the biceps anchor and therefore may be misinterpreted as a labral tear [29]. Despite that, it must be emphasized that high signal extending anterior and posterior to the biceps anchor is the hallmark of SLAP lesions.

**Fig. (4):**
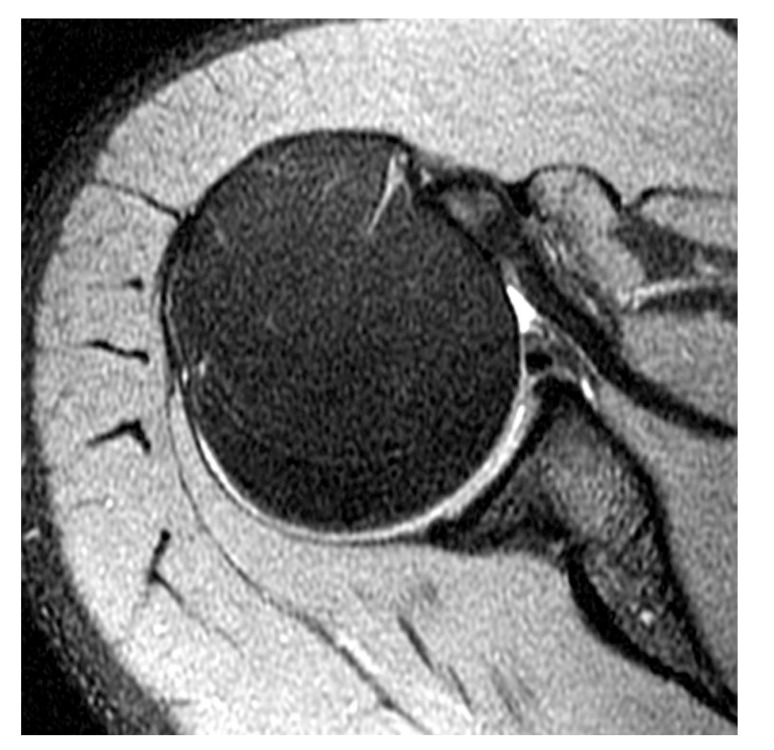
Sublabral foramen. (**A**) Axial fat-suppressed T2-weighted image of a 29 year-old male athlete with smoothly marginated, full-thickness separation of the labrum isolated to the anterosuperior quadrant.

The sublabral foramen occurs only in the upper anterior quadrant of the glenoid and does not involve the LHB insertion (Fig. **4**). The sublabral foramen appears as a smoothly marginated separation of the anterosuperior labrum from the underlying glenoid (over a range from approximately 3 to 11 mm cranial to caudal) and has a prevalence of approximately 11% [23].

**Fig. (5):**
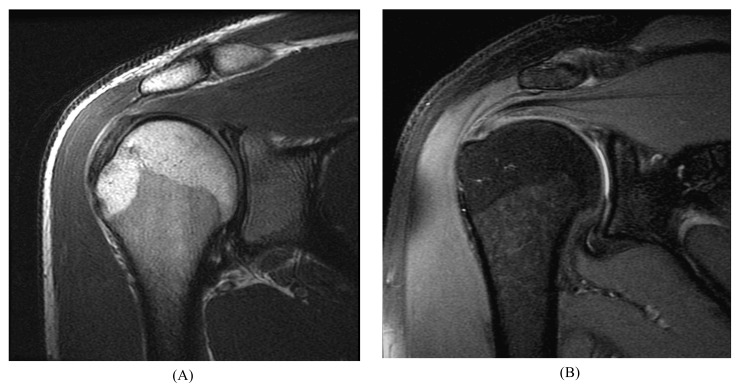
SLAP tear. Laterally curved linear signal located posterior to the biceps anchor in (**A**) a 22 year-old collegiate baseball pitcher, on a coronal intermediate-weighted image and (**B**) a 27 year-old overhead athlete, on a coronal fat-suppressed T2-weighted image.

**Fig. (6):**
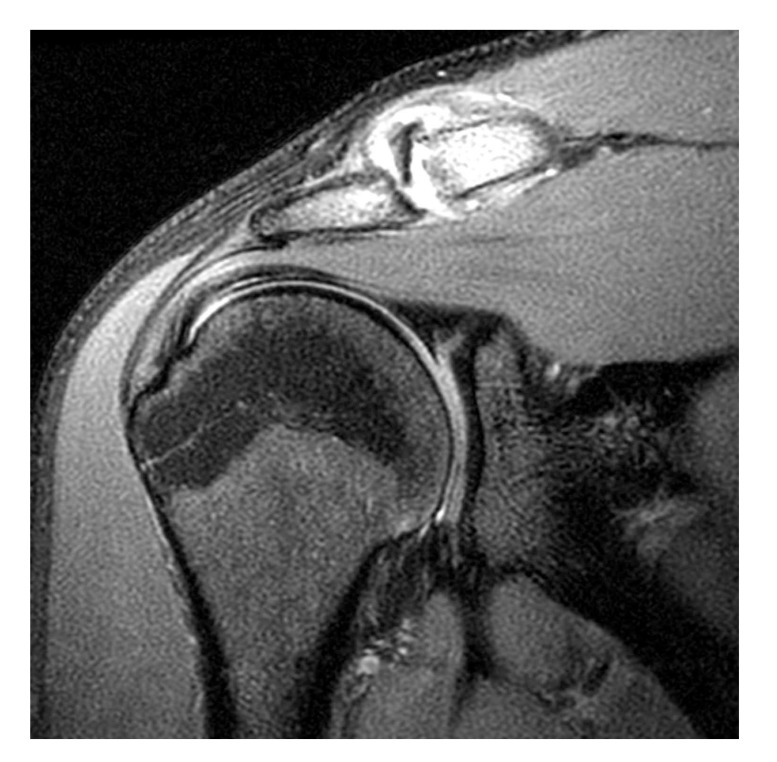
SLAP tear. Branching linear signal located posterior to the biceps anchor in a 19 year-old collegiate athlete on a coronal fat-suppressed T2-weighted image. Also note the presence of distal clavicle erosion and edema in this athlete with distal clavicular osteolysis owing to weight lifting.

**Fig. (7):**
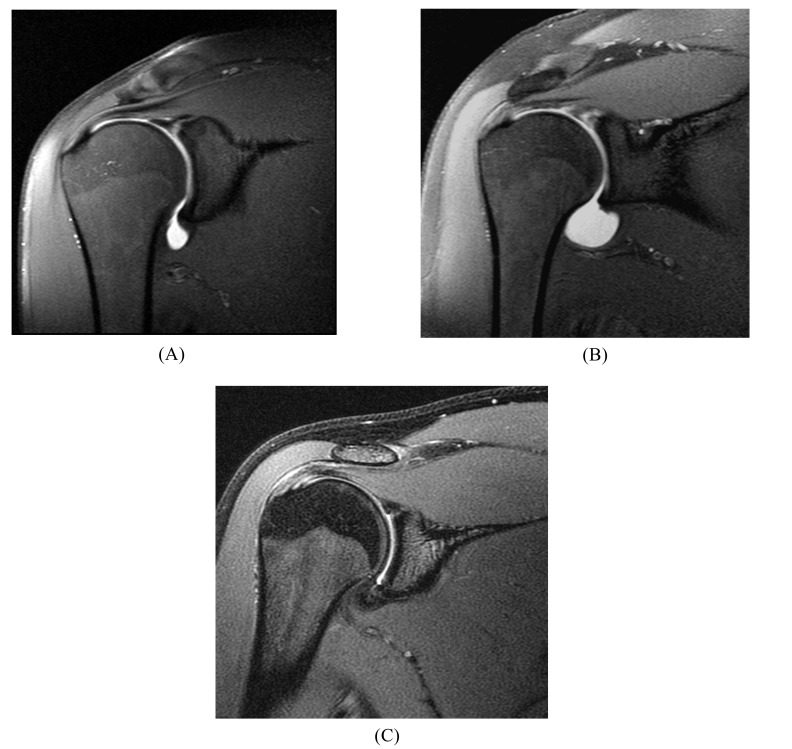
SLAP tear. Full-thickness detachment with linear signal separating the superior labrum from the glenoid rim in (**A**) a 21 year-old collegiate thrower, on a coronal fat-suppressed T1-weighted MR arthrogram, (**B**) a 28 year-old female athlete, on a coronal fat-suppressed T1-weighted MR arthrogram, and (**C**) a 19 year-old male athlete, on a coronal fat-suppressed T2-weighted MR image (without arthrography).

**Fig. (8):**
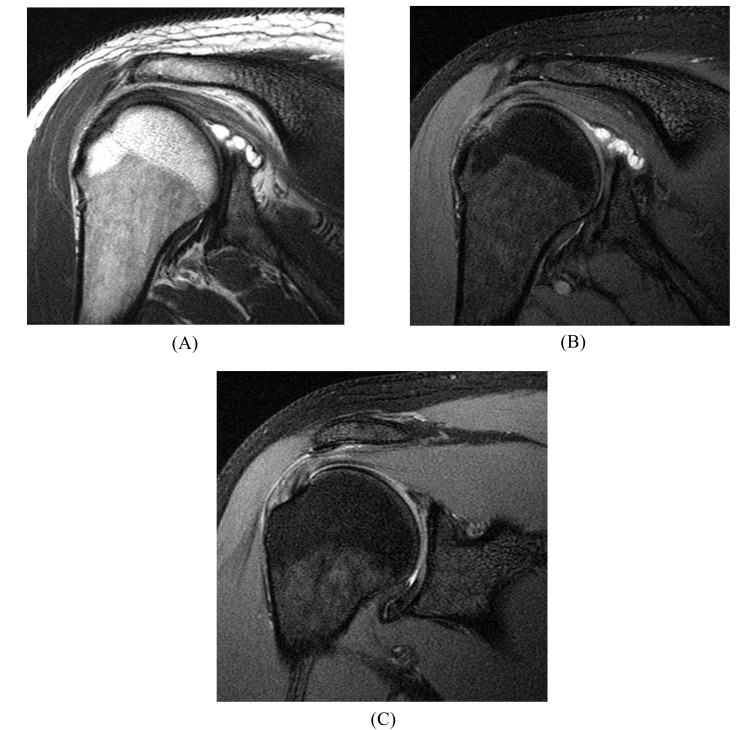
SLAP tear in a 39 year-old patient with a history of a negative c-spine MRI. Lobulated paralabral cyst at the posterosuperior glenoid rim due to a labral tear on (**A**) a coronal intermediate-weighted image and (**B**) a coronal fat-suppressed T2-weighted image. (**C**) At the adjacent superior labrum near the biceps anchor, there is subtle, laterally curved, linear signal in the labrum indicative of a SLAP tear (without arthrography).

Diagnostic criteria for a SLAP lesion by MR or MR arthrography most commonly include the following [29, 33]:

1. laterally curved, linear signal in the labrum on coronal imaging (Fig. **5**);
2. multiple or branching lines of high signal intensity in the superior labrum on coronal imaging (Fig. **6**);
3. full-thickness detachment with irregularly marginated high signal intensity and/or wide separation (e.g., width >2 mm on conventional MRI or 3 mm on MR arthrography) between the labrum and glenoid on coronal imaging (Fig. **7**);
4. a paralabral cyst extending from the superior labrum [34] (Fig. **8**).

Additional MR diagnostic criteria also have been proposed. The “sulcus score” has been developed to help differentiate between a superior labral sulcus versus a type II SLAP lesion [35]. The sulcus score quantifies the overall magnitude of the sulcus by accounting for both the sulcus depth and length. (The sulcus score is calculated by multiplying the depth (“grade”) of the sulcus by the length of the sulcus (the number of “hours” around the “clock” of the glenoid).

## 4 DISCUSSION

MR and MR arthrography with 1.5-T and 3-T scanners are currently the mainstays of imaging used to diagnose SLAP lesions prior to arthroscopy. Other modalities have been used to image the superior labrum, including CT arthrography which has been successfully utilized and ultrasound which has not been shown to be efficacious [36-38].

There is some evidence that the MR diagnosis of SLAP tears may be improved with provocative maneuvers, such as longitudinal traction of the arm [39] or positioning the shoulder in abduction and external rotation during imaging [40, 41]. The use of intra-articular contrast distends the joint similar to what occurs during arthroscopy and may improve the ability to detect SLAP lesions by filling any labral tear. Recent systematic reviews and meta-analyses conclude that MR arthrography is better than standard MR in detecting labral tears [42-44], with a higher sensitivity (80% vs. 63%) but similar specificity (91% vs. 87%) for SLAP tears [43].

*Is there improved diagnostic accuracy with 3-T MR imaging?* Although there is substantial heterogeneity in published studies and further research would be appropriate, the most recent pooled data suggests that improved diagnostic accuracy for SLAP tears results with 3-T compared with 1.5-T MR imaging [43, 44], with or without intra-articular contrast material (sensitivity, 78%–84% vs 79%–81% and specificity, 95%–99% *vs* 67%–84%) [43]. The U.S. Food and Drug Administration recently approved ultrahigh-field 7-T MR scanners for clinical use [45], but only preliminary studies have been performed on the shoulder [46].

## CONCLUSION

Continued technological advancements may result in improved accuracy of MR diagnosis of SLAP lesions. Regardless of any MR findings, however, physicians should be cautious when recommending surgery in the patient with a vague clinical picture. The patient’s history, physical exam, and imaging evaluation all should be considered together in making the decision to proceed with surgery.
